# Pressure-Induced Changes in the Crystal Structure and Electrical Conductivity of GeV_4_S_8_

**DOI:** 10.1021/acs.chemmater.3c02488

**Published:** 2024-03-21

**Authors:** Yuejian Wang, Zhiwei Shen, Dongzhou Zhang, Lin Wang, Vladimir Tsurkan, Lilian Prodan, Alois Loidl, Bishal B. Dumre, Sanjay V. Khare

**Affiliations:** ‡ Physics Department, Oakland University, Rochester, Michigan 48309, United States; # Center for High-Pressure Science (CHiPS), State Key Laboratory of Metastable Materials Science and Technology, Yanshan University, Qinhuangdao, Hebei 066004, China; ¶ Partnership for Extreme Crystallography, University of Hawaii at Manoa, Honolulu, Hawaii 96822, United States; § Experimental Physics V, Center for Electronic Correlations and Magnetism, University of Augsburg, Augsburg 86135, Germany; † Institute of Applied Physics, Moldova State University, MD-2028 Chisinau, Republic of Moldova; & Department of Physics and Astronomy, and Wright Center for Photovoltaics Innovation and Commercialization (PVIC), University of Toledo, Toledo, Ohio 43606, United States

## Abstract

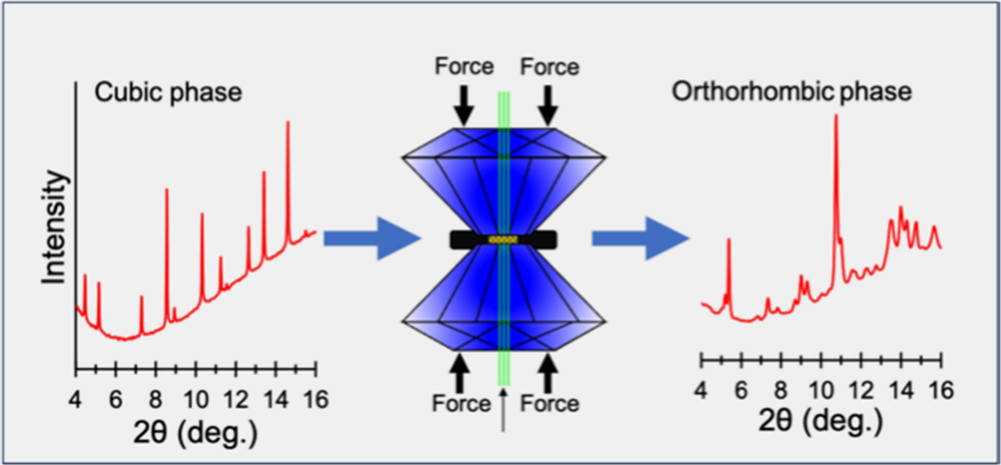

Lacunar spinels, represented by AM_4_X_8_ compounds (A = Ga or Ge; M = V, Mo, Nb, or Ta; X = S or Se), form a unique group of ternary chalcogenide compounds. Among them, GeV_4_S_8_ has garnered significant attention due to its distinctive electrical and magnetic properties. While previous research efforts have primarily focused on studying how this material behaves under cooling conditions, pressure is another factor that determines the state and characteristics of solid matter. In this study, we employed a diamond anvil cell in conjunction with high-energy synchrotron X-ray diffraction, Raman spectroscopy, four-point probes, and theoretical computation to thoroughly investigate this material. We found that the structural transformation from cubic to orthorhombic was initiated at 34 GPa and completed at 54 GPa. Through data fitting of volume vs pressure, we determined the bulk moduli to be 105 ± 4 GPa for the cubic phase and 111 ± 12 GPa for the orthorhombic phase. Concurrently, electrical resistance measurements indicated a semiconductor-to-nonmetallic conductor transition at ∼15 GPa. Moreover, we experimentally assessed the band gaps at different pressures to validate the occurrence of the electrical phase transition. We infer that the electrical phase transition correlates with the valence electrons in the V_4_ cluster rather than the crystal structure transformation. Furthermore, the computational results, electronic density of states, and band structure verified the experimental observation and facilitated the understanding of the mechanism governing the electrical phase transition in GeV_4_S_8_.

## 1 Introduction

Lacunar spinels with a chemical composition of AM_4_X_8_ (A = Ga or Ge; M = V, Mo, Nb, or Ta; X = S or Se) represent a large class of ternary chalcogenide compounds. Within this family of materials, two specific compounds, GaV_4_S_8_ and GeV_4_S_8_, have garnered significant attention due to their unique electrical and magnetic properties. These properties are closely linked to the presence of weakly bonded molecular units, cubane (M_4_X_4_)^*n*+^ and tetrahedral (AX_4_)^*n*−^ clusters, which are arranged in a lattice network reminiscent of NaCl.^1−15^ Due to this reason, the majority of studies predominantly focus on the electromagnetic properties of these two materials under cooling conditions, and a plethora of unusual phenomena have been observed.^2−4,6,7,9,13,15,16^ For example, the Néel-type skyrmion lattice in a bulk crystal was discovered in GaV_4_S_8_ for the first time, even though this structure had been theoretically predicted decades ago.^8^

As Mott insulators with strongly correlated properties, these materials exhibit remarkable coupling between spin, orbital, and lattice degrees of freedom. Consequently, the phase transitions, including electric, magnetic, and crystal structure changes, are often correlated. In the case of GeV_4_S_8_, experiments revealed two successive structural transformations at low temperatures. The material initially transitions from the starting cubic phase (space group *F*4̅3*m*) to an orthorhombic structure (space group *I*4̅*m*2) at around 30 K and then to another orthorhombic phase (space group *Imm2*) at approximately 17 K.^7,15,17^ This transition from cubic to orthorhombic symmetry is induced by the Jahn–Teller distortion. From a magnetic standpoint, GeV_4_S_8_ is paramagnetic under ambient conditions due to the presence of a 3-fold degenerate state with two unpaired electrons in the highest orbital of the V4 tetrahedral unit.^18^ As the material is cooled, antiferromagnetic ordering emerges below 13–18 K, coinciding with the structural shift from *I*4̅*m*2 to *Imm2*.^7,15,17^ Both phases share the same orthorhombic symmetry, with the primary distinction being the elongation of the longest V–V bond in *Imm2* compared to that in *I*4̅*m*2. In essence, *Imm2* represents a distortion of *I*4̅*m*2. Additionally, at low temperatures, the material exhibits coexistence between antiferromagnetic ordering and ferroelectric polarization, classifying GeV_4_S_4_ as a multiferroic material.^2^

In addition to cooling, compression provides another valuable means of manipulating and revealing the physical properties of strongly correlated materials due to the aforementioned remarkable coupling among lattice, electronic, and spin degrees of freedom in these systems. Compression allows for the adjustment of lattice spacing and can induce structural transitions, thereby modifying the material’s electromagnetic characteristics. For instance, compression has been found to induce superconductivity in certain lacunar spinel compounds.^19^ The Jahn–Teller phenomena, typically observed at low temperatures, have been detected in conventional spinel materials such as CuWO_4_, MnCr_2_O_4_, NiCr_2_O_4_, and ZnCr_2_S_4_ under high pressure conditions at room temperature.^20−22^ We conducted a comprehensive high-pressure study on GaV_4_S_8_, which has led to several interesting discoveries. These include the identification of the crystal structure of its high-pressure phase, determination of the bulk moduli for both the initial and high-pressure phases, and the observation of a pressure-induced electrical phase transition from insulator to metallic conductor.^10^ Despite the significance of high-pressure studies, we were unable to locate any existing literature on the high-pressure behavior of GeV_4_S_8_.

In this study, we employed high-pressure techniques in conjunction with synchrotron X-ray diffraction, Raman spectroscopy, and electrical resistivity measurements using a four-probe approach to systematically investigate GeV_4_S_8_ under cold compression conditions. Our research encompassed the detection of pressure-induced phase transitions, determination of the crystal structure of the high-pressure phase, and calculation of the bulk moduli for each phase. High-pressure Raman spectra were employed to complement and confirm the structural transformations identified via powder X-ray diffraction. Additionally, we explored the changes in electrical conductivity for GeV_4_S_8_ under high pressure at room temperature and under heating conditions, allowing us to construct an electrical phase diagram as a function of pressure. Finally, through the analysis of alterations in lattice parameters, bonding lengths in response to pressure, and the density of states and electronic band structure, we sought to clarify the electrical phase transition under compression.

## 2 Experimental Methods

The starting materials for this study include single crystalline and polycrystalline GeV_4_S_8_, with the synthesis details reported elsewhere.^4^ In summary, polycrystalline GeV_4_S_8_ was synthesized through solid-state reactions using high-purity elements: Ge (99.9999%), V (99.95%), and S (99.9999%). GeV_4_S_8_ single crystals were subsequently grown from the polycrystalline materials using the chemical transport reaction method with iodine serving as a transport agent. The growth process took place in a two-zone furnace with a temperature gradient of 860–820 °C. Perfect single crystals with dimensions up to 4 mm were achieved after 2 months of transport. The powders investigated in this research were produced by crushing and grinding the single crystals. Synchrotron X-ray data confirmed the high purity of the sample materials with no detectable impurities. High-pressure conditions were achieved by using a diamond anvil cell with a culet size of 300 μm. A rhenium foil was used as the gasket material, in which a sample chamber hole with a diameter of 130 μm and a depth of approximately 45 μm was created by using an electrical discharge machining system. Sample pressures were calibrated using ruby fluorescence.^23^ To minimize the pressure gradient across the sample, we utilized helium gas and silicon oil as the pressure-transmitting media (PTM) for high-pressure X-ray diffraction and Raman measurements, respectively.^24,25^ A gas-loading system at GSECARS of Argonne National Laboratory was employed to introduce helium gas into the sample chamber within the diamond anvil cell.^26^ The synchrotron powder X-ray diffraction measurements were conducted at the beamline 13-BM-C of the Advanced Photon Source (APS) at Argonne National Laboratory.^27^ During these X-ray diffraction measurements, the sample was automatically compressed by using a membrane system. The incident monochromatic X-ray beam had a wavelength of 0.434 Å and was focused to an approximate size of 12 μm (horizontal) × 18 μm (vertical), as determined by measuring the full width at half-maximum of the beam spread. X-ray diffraction patterns were collected by using a Pilatus 1 M area detector positioned 198 mm away from the sample. Prior to measurement, an ambient diffraction pattern was obtained from powdered SRM660a LaB_6_ to calibrate the distance between the sample and the area detector, as well as the tilt of the area detector. The collected X-ray powder Debye rings were converted into conventional diffraction patterns using Dioptas software, and these patterns were subsequently processed using GSAS-II software based on the Rietveld refinement method to determine the lattice parameters and bond lengths of GeV_4_S_8_ at each pressure point.^28,29^ VESTA was used to visualize and depict the crystal structures of the sample.^30^

High-pressure Raman measurements were carried out using an inVia Renishaw Raman system equipped with a green laser (532 nm wavelength) and a grating of 2400 g/cm. The Raman shift resolution was 1 cm^–1^. For electrical resistivity measurements under pressure, a standard four-probe system was integrated into a diamond anvil cell, and no PTM was utilized. To maintain proper insulation between the electrodes, a layer of epoxy mixed with cubic boron nitride was applied to the surface of the steel gasket, on which the four-probe system was mounted. Platinum electrodes made direct contact with the sample and were connected to a 4050 Keithley digital multimeter via copper wires. The resistance of the sample under varying pressures was calculated using the van der Pauw method, with input provided by current and voltage measurements obtained through the Keithley digital multimeter.^31^ The great conductivity of platinum and copper, as well as the direct contact between the sample and the electrodes, greatly minimized the measurement uncertainty. Single crystals of GeV_4_S_8_ were used to perform the high-pressure Raman/electrical resistivity experiments.

During the high pressure measurements, we selected a position on the sample as close to the ruby sphere as possible for data collection. Pressure was measured both before and after collecting data, such as the X-ray profile. The sample pressure was then determined by averaging those two values. Despite fluctuations in uncertainty with pressure, it typically remains below 1 GPa.

## 3 Computational Methods

All density functional theory (DFT) calculations were performed using the Vienna Ab initio Simulation Package (VASP).^32−35^ The Perdew–Burke–Ernzerhof (PBE)^36,37^ exchange-correlation functional was employed within the Generalized Gradient Approximation (GGA) framework to conduct the Projector-Augmented-Wave (PAW) method^38,39^ in the majority of the computations. The calculations included semicore electrons in addition to the outer core, specified by “V_sv”, “Ge_d”, and “S” pseudopotentials in VASP. Plane waves of an energy cutoff of 500 eV were utilized, and a Γ-centered k-point mesh was applied, incorporating 4000 k-points per reciprocal atom (KPPRA) in the calculations.^40−42^ Structural relaxation of each atom continued until the forces were below 0.01 eV/Å. The convergence in energy in electronic iterations was set to 10^–6^ eV/atom, employing a Gaussian smearing of width of 0.05 eV.^43−45^ Initially, crystal structures were sourced from Materials Project.^46^

Electronic band gaps tend to be underestimated when utilizing the Generalized Gradient Approximation (GGA) and Local Density Approximation (LDA) exchange-correlation functionals.^47^ To address this limitation, we employed the Heyd–Scuseria–Ernzerhof hybrid functional (HSE06), which combines 25% of the exact exchange from Hartree–Fock theory and 75% of the exchange from GGA, to determine the electronic density of states (DOS).^48,49^ In semiconductors and insulators, the HSE06 hybrid functional has demonstrated an improved accuracy in predicting results compared to GGA.^47,50−52^

We initiated our simulation by utilizing the conventional unit cells of GeV_4_S_8_ in cubic and orthorhombic crystal structures obtained from Materials Project with Material IDs mp-8688 and mp-1103812, respectively.^46^ Subsequently, we performed structural relaxation and assessed the magnetism for both structures. Due to computational constraints, we then transformed both structures into primitive unit cells. Following this, we subjected both structures to various pressure conditions and computed their structural, energetic, and electronic properties.

## 4 Results and Discussion

**Figure 1:**
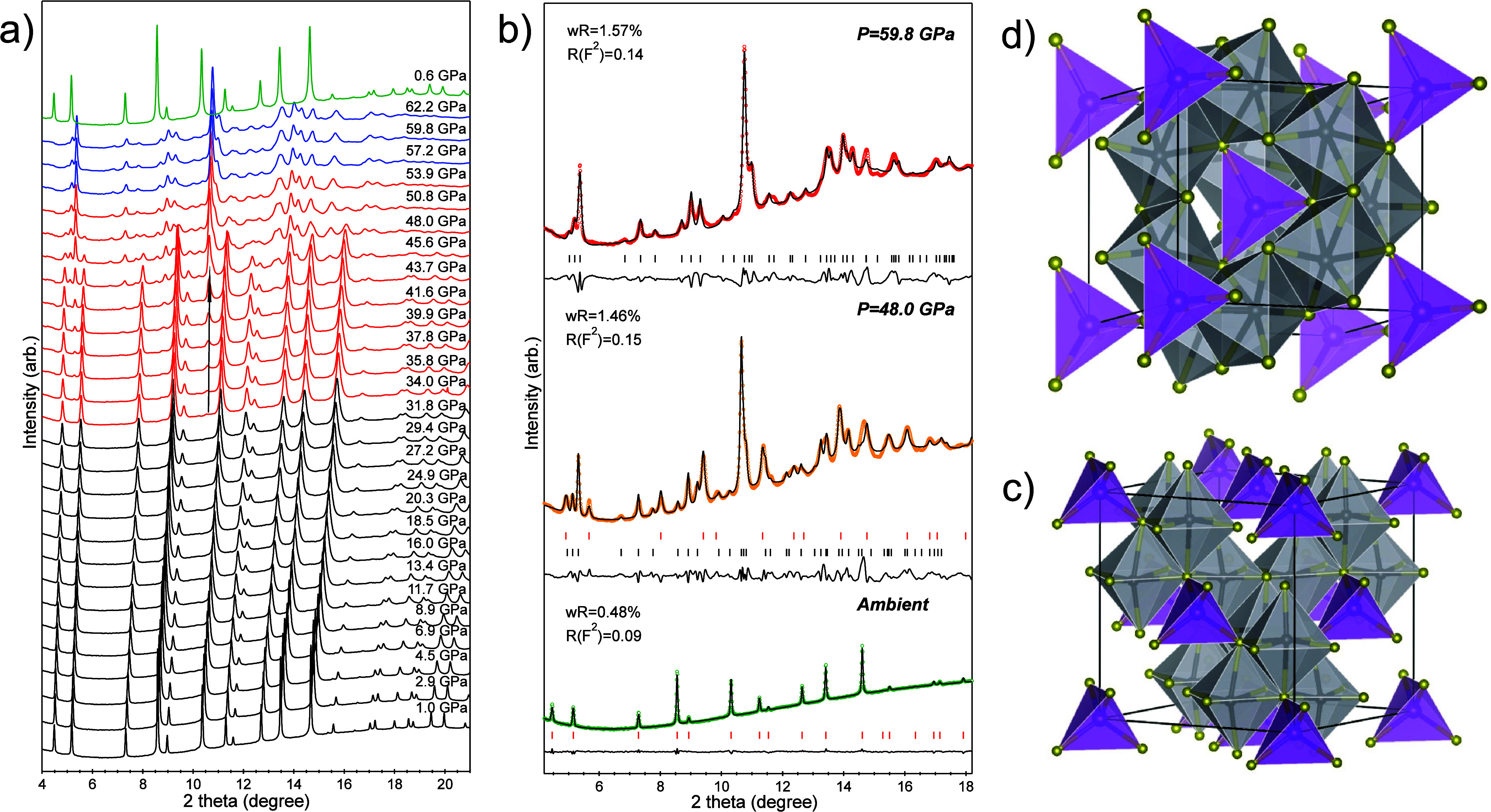
(a) X-ray diffraction patterns of GeV_4_S_8_ under high pressure. In this figure, black, red, and blue represent the patterns corresponding to the cubic phase, a mixture of cubic and orthorhombic phases, and the orthorhombic structure, respectively. The top green pattern represents the data collected after releasing the pressure to 0.6 GPa. (b) Examples of refinements for the X-ray diffraction patterns. In each case, the collected patterns are shown as open circles, while the calculated patterns are depicted as solid curves. The difference between those two is represented by the curve located beneath the diffraction patterns. We selected three pressure points: at the bottom (ambient conditions), in the middle (48.0 GPa), and at the top (59.8 GPa), representing the pure cubic phase, a mixture of cubic and orthorhombic phases, and the pure orthorhombic structure, respectively. The visional representations of the unit cells of the cubic structure (c) and orthorhombic structure (d) of GeV_4_S_8_, consisting of two types of polyhedra, VS_6_ and GeS_4_, where Ge, V, and S atoms are visualized by purple, black, and yellow balls, respectively.

In the initial stage of our investigation, we characterized the sample under ambient conditions using synchrotron X-ray diffraction. The obtained pattern exhibited clear signals from GeV_4_S_8_ without any noticeable impurities. Furthermore, we performed Rietveld refinement on the X-ray pattern, as depicted in Figure 1, which allowed for precise indexing. The analysis revealed a face-centered cubic structure with an *F*4̅3*m* space group (no. 216) and a lattice parameter of 9.6574 Å. These results are consistent with prior studies,^4,18^ and the lattice parameter is similar to that of GaV_4_S_8_.^10,53^ Subsequently, we placed the powdered sample within a diamond anvil cell to collect high-pressure X-ray diffraction profiles. As illustrated in Figure 1a, increasing pressure led to a gradual shift of the X-ray peaks toward higher angle positions. This shift occurred due to the compression-induced reduction in the atomic layer spacing. Apart from these peak shifts, no abrupt changes were observed until a new peak emerged at ∼10.5° around 34 GPa, as shown in Figure 1a. This appearance signified the initiation of a phase transition, which occurred gradually over a broad pressure range spanning from 34 to 54 GPa. The sample eventually transformed to the pure high-pressure phase at 57.2 GPa. Additionally, the X-ray diffraction patterns displayed broad, weak, and asymmetric peaks, suggesting that the high-pressure phase contained disordered fine grains, possibly with partial amorphization. The high-pressure phase remained stable up to 62.2 GPa with no further transformations. Consequently, we identified three distinct regions within the sample’s composition: a pure cubic phase from ambient pressure to 34 GPa, a mixture of cubic and high-pressure phases between 34 and 54 GPa, and a pure high-pressure phase above 57.2 GPa up to 62.2 GPa.

By comparing the high-pressure behavior of GeV_4_S_8_ with that of GaV_4_S_8_, we propose an orthorhombic structure (space group *Imm*2, no. 44) for the high-pressure phase of GeV_4_S_8_, as the X-ray profiles of these two materials under high pressure exhibit striking similarities.^10^ We attempted to fit the experimental X-ray data, which included the high-pressure phase of GeV_4_S_8_, and the excellent fitting results, as shown in Figure 1b, confirmed that the high-pressure phase of GeV_4_S_8_ crystallizes in an orthorhombic structure, matching the symmetry of its low-temperature polar phase.^4^ Therefore, the phase transition scenarios of GeV_4_S_8_ under high pressure and low temperature are quite similar to the material transforming into an orthorhombic structure from the starting cubic phase. In other words, compression and cooling have the same thermodynamic impact on the crystallization of this material.

Releasing the pressure to 0.6 GPa, the X-ray pattern reverts to the starting cubic phase, indicating that the high-pressure phase is unquenchable. Upon comparing the initial ambient pattern with the recovered one, it becomes evident that after experiencing the compression and decompression, the constituents of the sampling materials have been squeezed/cut/torn/broken into smaller grains, as indicated by the broader peaks in the recovered X-ray pattern.

The crystallographic images of the starting and high-pressure phases of GeV_4_S_8_ are illustrated in Figure 1c,d, showing that both structures are substantially similar, featuring two types of polyhedra: VS_6_ octahedra and GeS_4_ tetrahedra with a 90° angle between lattice parameters. Under compression, the initial cubic phase with face-centered GeS_4_ tetrahedra undergoes distortion, transitioning into an orthorhombic structure where lattice parameters *a* ≠ *b* ≠ *c*, and body-centered GeS_4_ tetrahedra become prevalent. Table S1 presents the crystallographic data for both phases. Due to the unquenched nature of the high-pressure orthorhombic phase, the crystallographic data listed in the table were from the sample under ∼60 GPa, at which the sample completely transformed into the orthorhombic phase, so the high purity ensures the precision of the data.

**Figure 2:**
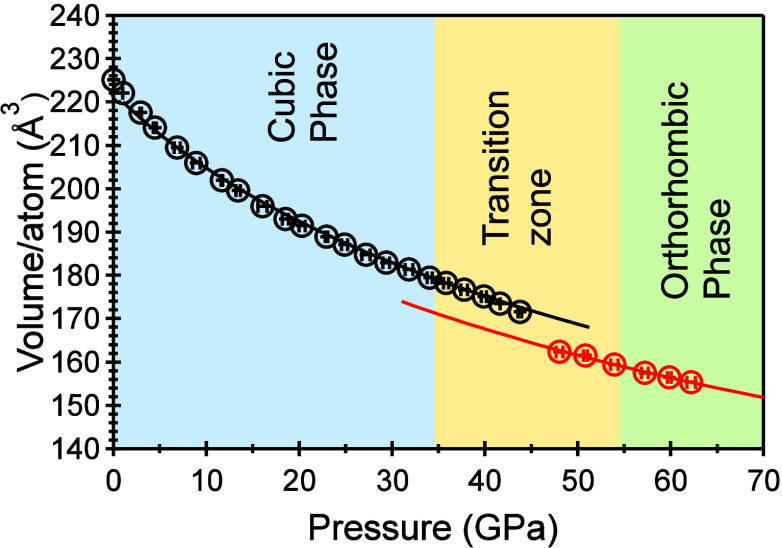
Data fitting of volume occupied by individual atom vs pressure. The volumes derived from the cubic and orthorhombic phases are represented by black and red circles, respectively. The error bars, which are shorter than the sizes of the circles, indicate the uncertainties. The solid curves represent the fitting results obtained using the second-order Birch–Murnaghan equation of state.

The X-ray patterns were refined by using GSAS-II to determine the volume occupied by each atom under varying pressures. Subsequently, the bulk moduli for the cubic and orthorhombic structures were obtained by fitting the pressure–volume (*P*–*V*) data set to a second-order Birch–Murnaghan equation of state,^54,55^
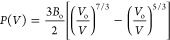
in which *B*_o_ represents the bulk modulus, *V*_o_ stands for the volume under ambient conditions, and *V* refers to the volume at a given pressure, as shown in Figure 2. During the fitting by using EosFit7GUI,^56^ the derivative of the bulk modulus with respect to pressure was fixed to 4, and the least-squares fitting yielded bulk moduli of 105 ± 4 GPa for the cubic phase and 111 ± 12 GPa for the orthorhombic phase. The volumes per atom at 0 GPa were determined to be 222 ± 1 A^3^ for the cubic phase and 211 ± 4 A^3^ for the orthorhombic phase. These results indicate that these two phases have very close stiffness but are both softer than corresponding phases of GaV_4_S_8_.^10^ Notably, the orthorhombic phase of GeV_4_S_8_ is over two times softer than that of GaV_4_S_8_.^10^ It is important to note that the uncertainty in the bulk modulus was determined solely from the fitting process, and it does not account for the propagation of uncertainties from pressure and volume values.

**Figure 3:**
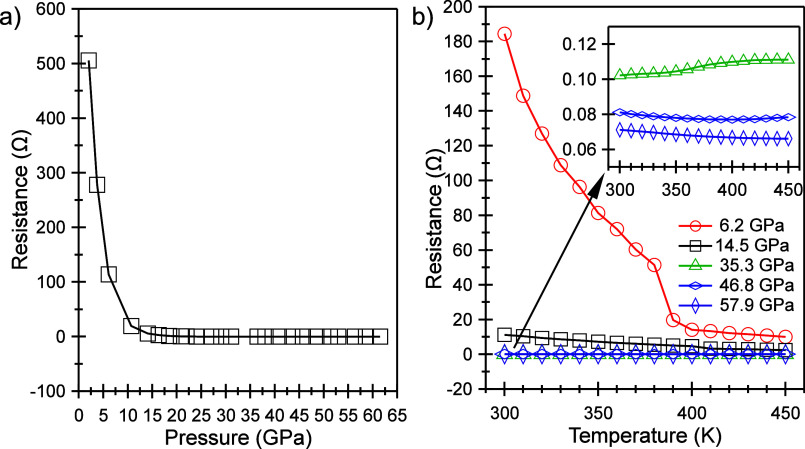
(a) Electrical resistance of GeV_4_S_8_ as a function of pressure at room temperature. (b) Electrical resistance versus temperature at five pressure points.

The electrical transport properties of a single crystal of GeV_4_S_8_ under high-pressure conditions were characterized at room temperature. Under ambient conditions, the electrical resistance, which is approximately 500 Ω, suggests that this compound is a semiconductor. The same electrical property has been found in GaV_4_S_8_^10^ as well as in other ternary chalcogenides.^5,7,19^ As shown in Figure 3a, starting from ambient pressure, the electrical resistivity of GeV_4_S_8_ decreases rapidly, almost linearly, until the pressure reaches around 10 GPa. Thereafter, it enters a stable region in which its resistivity, slightly above 0 Ω, is almost independent of the pressure value. Beyond 15 GPa, the low resistivity indicates that the band gap has closed, and GeV_4_S_8_ has fully transformed into an electrical conductor. This transition pressure from semiconductor to conductor, which occurs between 10 and 15 GPa, is different from the crystal structural transition pressure, which is at 34 GPa. This suggests that the change in electrical properties under high pressure may not be solely due to alterations in the crystal structure of GeV_4_S_8_.

Furthermore, to determine whether the conducting phase of GeV_4_S_8_ exhibits metallic behavior, we subjected this material to a fixed pressure while varying temperatures and measured its in situ electrical resistance, as shown in Figure 3b. At 6.2 GPa, a significant drop in resistance occurs in the range of room temperature to around 400 K. at higher pressures, the resistance decreases slowly with the temperature, which is not characteristic of a metal undergoing heating. Therefore, this material remains a nonmetallic conductor, at least up to 57.9 GPa. In contrast, despite having the same crystal structure under ambient conditions and undergoing a similar crystal structural transformation under high pressure, GaV_4_S_8_ transitions from a semiconductor to a metal under high pressure.^10^ This evidence further illustrates the decoupling between the crystal structural transformation and the electrical phase transition induced by compression.

**Figure 4:**
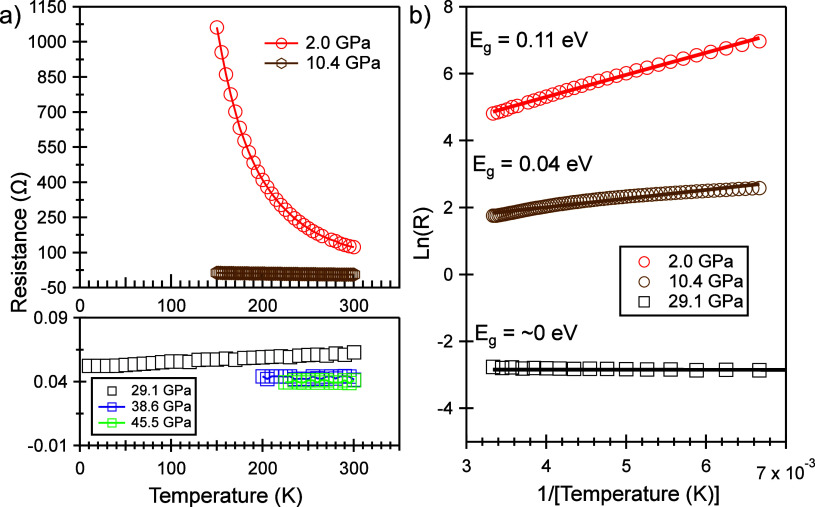
(a) Electrical resistance in GeV_4_S_8_ under cooling at 5 fixed pressure points. (b) Data plot of Ln(*R*) vs 1/*T*, fitted by the Arrhenius equation. From the linear fitting represented by the solid line, the band gap energy *E*_g_ was determined for each pressure point.

We also conducted resistance measurements on GeV_4_S_8_ under high-pressure and low-temperature conditions. As depicted in Figure 4a, at low pressures, such as 2.0 GPa, the resistance significantly increases as the temperature decreases, displaying the typical behavior of a semiconductor. However, at high pressures, such as 10.4, 29.1, 38.6, and 45.5 GPa, the resistance remains just slightly above 0 Ω and exhibits almost no dependence on pressure. Given its semiconductor nature, it is reasonable to assume a linear relationship between the natural logarithm of resistance, Ln(*R*), and temperature. Analyzing the slope of Ln(*R*) vs temperature allows us to determine the band gap energy at various pressures, as depicted in Figure 4b. At ambient pressure, from our computation, the initial cubic structure has a band gap of 0.27 eV, which is consistent with the previous experimental determination.^4^ Hence, it is evident, as shown in Figure 4b, that the band gap narrows with increasing pressure and ultimately reaches zero. This observation further confirms that GeV_4_S_8_ undergoes a transition from a semiconductor to a conductor under compression.

**Figure 5:**
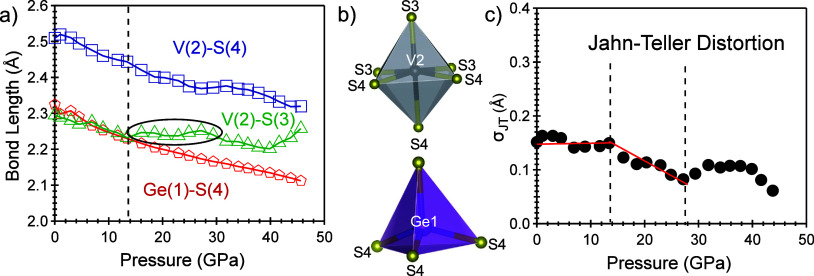
(a) Bond length of the cubic phase as a function of pressure. The dashed straight line marks the beginning of the flattening, enclosed in a circle, of V(2)–S(3) versus pressure. (b) Graphic representation of octahedra, VS_6_ (top) and GaS_4_ (bottom). (c) Jahn–Teller distortion, σ_JT_, versus pressure.

From the measurements described above, we observed that the transition from a semiconductor to a conductor occurred at approximately 15 GPa (as shown in Figure 3a). This transition pressure is notably lower than the pressure at which the structural transition from cubic to orthorhombic, as probed by X-ray diffraction, occurs. To address this inconsistency, we closely examined the changes in bond lengths under pressure, as depicted in Figure 8a. Under ambient conditions, the V(2)–S(4) bonds are longer than the V(2)–S(3) bonds, leading to the V atom not being situated at the geometric center of the VS_6_ octahedron, as illustrated in Figure 8b. As pressure increases, all of the bonds shorten, but starting from approximately 14 GPa, the V(2)–S(3) bond becomes nearly constant in length as circled in Figure 5a, , causing the V atom to gradually move toward the center of the VS_6_ octahedron. This motion of the V atom, reducing the distortion of the VS_6_ octahedron, may give rise to localized electronic structure changes within the V_4_S_4_ cluster, resulting in significant alterations in transport properties.^5,10^ In the electrical conductivity measurement, we observed that the material undergoes a phase transition from a semiconductor to a conductor at around 15 GPa, which closely aligns with the pressure point mentioned earlier in this paragraph. Therefore, this electrical phase transition can be attributed to the significant changes in the atomic bond lengths under compression.

To quantitatively evaluate the Jahn–Teller effect under compression, the Jahn–Teller distortion σ_JT_ has been characterized by the following equation^57−59^
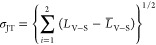
where *L*_V–S_ represents the bond lengths between atoms V and S, such as *L*_V(2)–S(3)_ and *L*_V(2)–S(4)_, while *L̅*_V–S_ is the average distance between V and S in the VS_6_ octahedron under various pressures. As shown in Figure 5c, from ambient pressure up to ∼13 GPa, the JT distortion is relatively stable with a slight fluctuation, but starting from ∼13 GPa, the JT distortion continuously decreases with pressure up to ∼27 GPa, and then it changes nonlinearly. Therefore, two critical pressure points were observed, ∼13 and ∼27 GPa. The first pressure point, 13 GPa, aligns very well with the transition pressure point from a semiconductor to a conductor, which validates our preceding discussion. The pressure point of ∼27 GPa may be related with other changes in the materials, such as magnetic dynamics, but it is not the primary focus in the present study.

We calculated the electronic density of states (DOS) using HSE06 for a primitive unit cell to gain further insight into the changes in the electrical conductivity of GeV_4_S_8_ under high pressures. As illustrated in Figure S1 in the Supporting Information, the initial cubic structure exhibits semiconductor behavior with a band gap of 0.27 eV. Under compression, the band gap disappears in the orthorhombic structure at a pressure of 60 GPa, transforming the material into a conductor, aligning with experimental observations. Particularly, we observe that the gap in the bands has shifted above the Fermi level in the orthorhombic structure at a pressure of 60 GPa, which implies that as we observed in the experimental measurements, the high-pressure phase is a nonmetal conductor because the valence and conduction bands do not fully merge. Upon careful scrutiny of the electronic DOS for the three elements in the material, we found that the DOS of vanadium dominates around the Fermi level, both in the initial cubic phase and in the high-pressure orthorhombic phase. This implies that the motions and displacements of vanadium ions in the lattice structure or the shortening and stretching of chemical bonds between vanadium and its neighboring ions play a critical role in the electronic structure of the material under high pressure. A similar phenomenon has been observed in the X-ray diffraction measurement. As depicted in Figure 5a, the bonding length of V(2)–S(3) remains relatively unchanged starting at 14 GPa and even stretches instead of shortening above 40 GPa. This anisotropic behavior causing the Jahn–Teller effect could serve as a catalyst for the transition in the electronic structure. Similarly, the electronic band structure, presented in Figure S2 in the Supporting Information and calculated using GGA for a primitive unit cell, reveals notable characteristics. Under higher pressure, in both crystal structures, it becomes apparent that the band lines with both up and down spins coincide, a phenomenon attributed to the applied pressure that effectively counters the repulsion of the spins.

**Figure 6:**
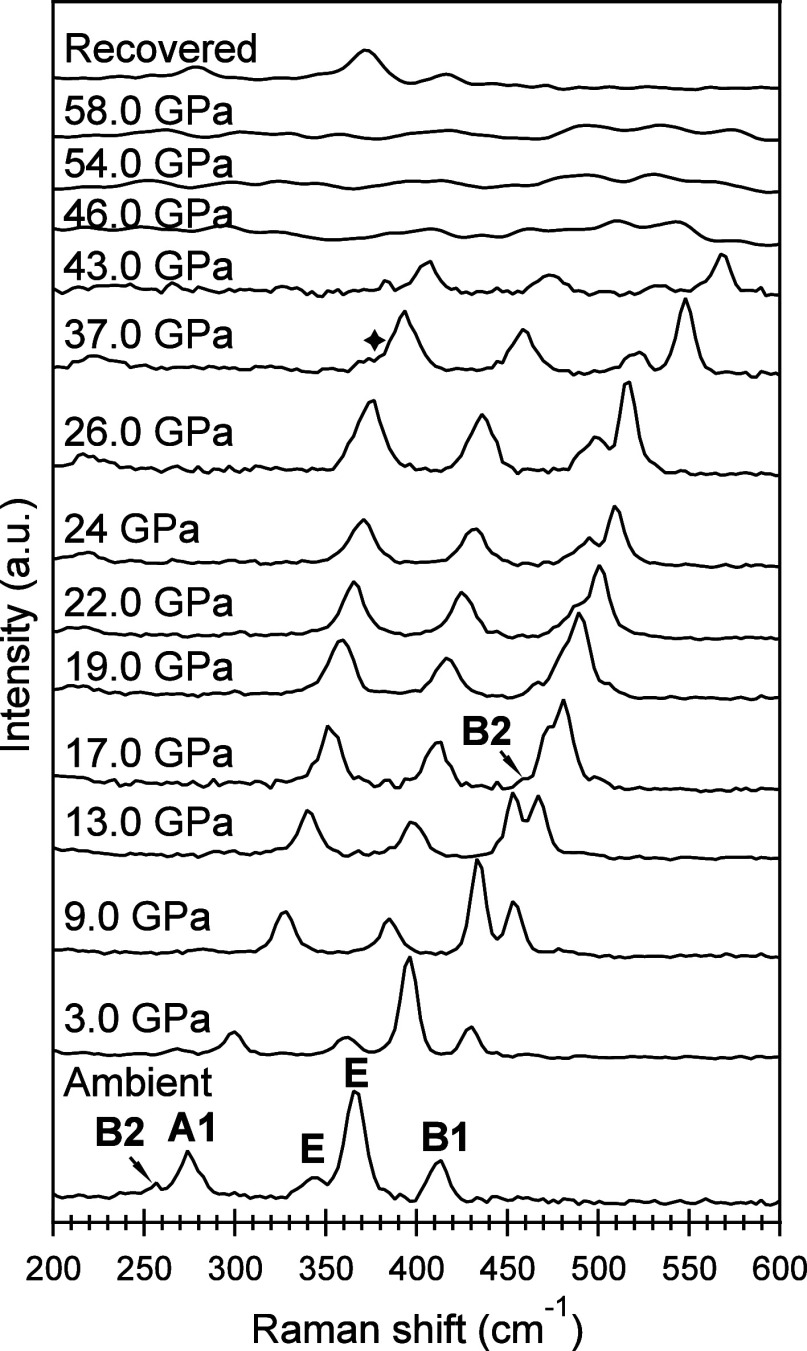
Raman spectra of GeV_4_S_8_ under high pressures.

High-pressure Raman measurements were conducted at room temperature by using a single crystal of GeV_4_S_8_, and the spectra are depicted in Figure 6. Previous study has performed full geometry optimization and density function theory to assign the phonon modes and predict the phonon frequencies (cm^–1^) and intensities. Therefore, here, we directly refer to those assignment results.^60^ Under ambient conditions, the Raman spectrum exhibits 5 active modes (B_2_, A_1_, B_1_, and two Es), which is consistent with another experimental study, although the number of modes is fewer than the theoretically predicted ones because of the weak signals maybe overshadowed by the background scattering.^60^ As the pressure increases, all of the peaks gradually shift to higher wavenumber positions, but the shifting rates vary significantly. For example, the E band, located at ∼365 cm^–1^ at ambient pressure, shifts to a higher wavenumber position more rapidly, while B1 shifts relatively slower. Consequently, the gap between these two modes becomes smaller with increasing pressure, and eventually, they overlap at around 19 GPa. Beyond this point, they switch positions in the spectra. The B_2_ mode, which was initially buried or overshadowed by other peaks, becomes visible, although the signal is very weak and only slightly above the background. Furthermore, the compression largely reduces the thickness of sample materials inside the pressure chamber as well as crushes the single crystal into fine particles, causing the peaks to become broader and weaker with increasing pressure. Starting from 46 up to 58 GPa, the highest pressure point during the measurement, all of the peaks evolve into broadly low-intensity bumps that are almost indistinguishable from the background scattering. Besides this, the presence of the conductor phase inside the sample during compression may further weaken the Raman features, a phenomenon similar to what has been observed in other lacunar spinels.^10^

**Figure 7:**
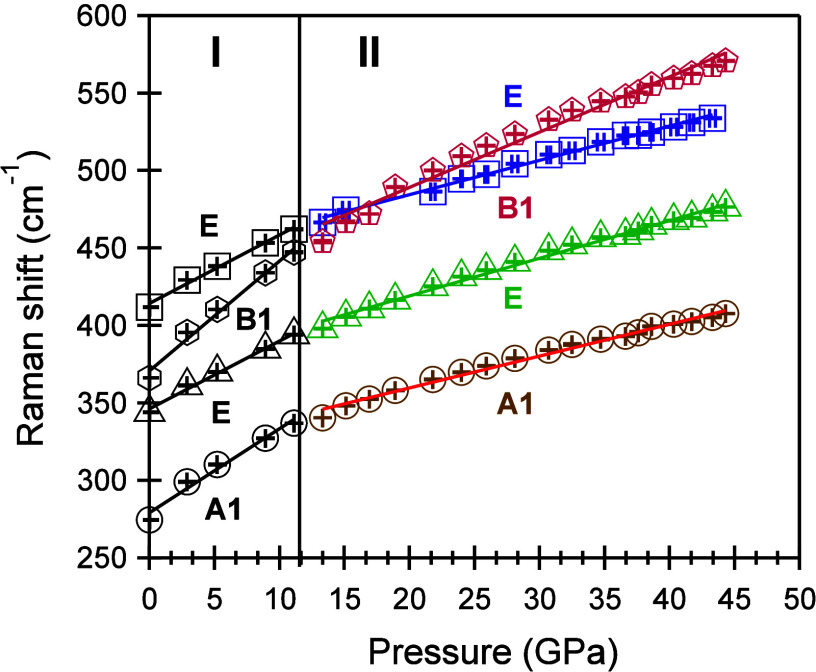
Peak positions of Raman modes plotted against pressure with linear data fitting represented by straight lines.

We determined the pressure coefficients, , as outlined in Table S2, through linear fitting of the Raman peak positions against pressures, as illustrated in Figure 7. This linear fitting reveals two distinct regions with a critical pressure point around ∼12 GPa. In region I, the pressure coefficients for A1, B1, and one of the E Raman modes closely range from 4.4 to 5.4 cm^–1^/GPa, while another E mode exhibits a significantly larger coefficient of 7.1 cm^–1^/GPa. Similarly, in region II, a noticeable inconsistency was observed, with one E mode showing a larger coefficient compared to those of the remaining Raman modes. This inconsistency hints a potential asymmetry in the force constants governing the phonon behavior. Clearly, the E mode’s smaller force constant makes it highly sensitive to applied pressure.

It is worth noting that the critical pressure point, approximately 12 GPa, which demarcates Figure 7 into two regions, aligns with the critical pressure point in Figure 5a,c and the pressure point for the electrical phase transition. Additionally, it influences the spring constant in the harmonic oscillator model representing the phonon features in the lattice structure. This alteration in the spring constant is refelcted in changes in the slope of the positions of the Raman peaks versus pressures, as illustrated in Figure 7.

Furthermore, we calculated the Grüneisen parameters (γ) using the formula , as listed in Table S2, where ω_o_ represents the peak position of the Raman mode under ambient conditions,  is the pressure coefficient, and *B*_o_ is the bulk modulus obtained earlier from the volume–pressure data fitting.^61^ One of the E modes in both regions I and II exhibits a larger γ value, consistent with the observation that this mode shifts more rapidly to larger wavenumber positions than do the other modes under compression. On average, the pressure coefficients of GeV_4_S_8_ are smaller compared to those of GaV_4_S_8_. Despite both spinels sharing the same crystal structure under ambient conditions and exhibiting a similar phase transition under high pressure, the different atoms, Ga and Ge, may induce different phonon responses to compression.^10^ These values are critically useful for calculating various other physical quantities, such as heat capacity and vibrational entropy.

**Figure 8:**
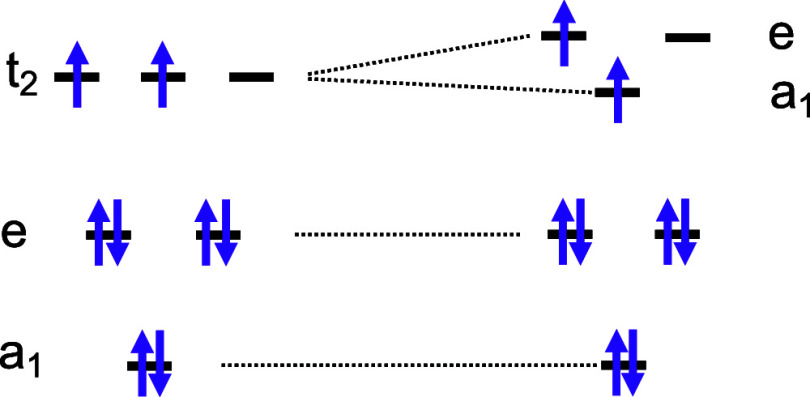
The transition of the molecular orbital of the V_4_ cluster unit in GeV_4_S_8_ under compression.

It is intriguing to draw a comparison between the two tetrahedral V_4_ cluster compounds, GaV_4_S_8_ and GeV_4_S_8_. Despite their striking similarities, the primary distinction lies in the incorporation of different elements (Ga and Ge) within their compositions. Ga and Ge, as neighboring elements in the periodic table with valence electrons of 4p^2^ and 4p^1^, respectively, may result in diverse valence states in these two compounds, thereby influencing their behavior under compression. Remarkably, both compounds exhibit the fcc structure under ambient conditions, undergoing a transformation to an orthorhombic structure within a similar pressure range of approximately 34 to 54 GPa. This implies that the structural transformation is likely associated with the dynamics of lattice planes, while the electronic density of states exerts a negligible influence. On the other hand, the electrical phase transitions in these two materials display disparities. Initially, both semiconductors undergo transformations into electrical conductors, while the transition pressures are different, 10–15 GPa in GeV_4_S_8_ versus 15–34 GPa in GaV_4_S_8_. We may turn to the electronic structures to find the reason for this discrepancy. First, as illustrated in Figure S1, the density of states near the Fermi level is predominantly influenced by V atoms. Consequently, these two compounds exhibit very similar band gaps, 0.3 eV in GeV_4_S_8_ and 0.24–0.30 eV in GaV_4_S_8_, as determined from resistivity measurements at ambient pressure.^4,9,62^ Second, the distinct numbers of valence electrons in Ga and Ge atoms result in different ionic formulas: Ga^3+^V_4_^3.25+^V_8_^2–^ and Ge^4+^V_4_^3+^V_8_^2–^ with seven and eight valence electrons per V_4_ cluster, respectively, in these two compounds. These valence electrons reside in three energy levels, namely, a_1_, 2-fold generated e, and 3-fold generated t_2_, as shown in Figure 8. During compression, due to the Pauli exclusion principle or the repulsion from the overlapping of the two valence electrons in the t_2_ orbitals, the Jahn–Teller effect caused by the compression-induced distortion of the V_4_ cluster can eliminate the 3-fold degeneracy. This tends to push those two electrons to two slightly different energy levels, which may facilitate the electron at the higher energy orbital hopping into the conduction band in GeV_4_S_8_.^5,7^ Therefore, compared to GaV_4_S_8_, which has only one electron in the t_2_ orbit initially, GeV_4_S_8_ is more likely to transform into an electrical conductor at a lower pressure point, as demonstrated in the experimental observations.

**Figure 9:**
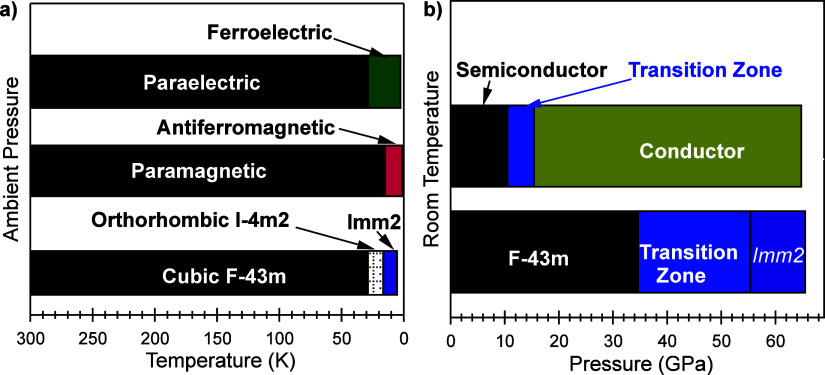
Phase diagram as a function of temperature (a) and pressure (b).

In summary, our X-ray diffraction study revealed that GeV_4_S_8_ undergoes a transformation from a cubic structure to an orthorhombic structure under high pressure at room temperature. This phase transition exhibited a sluggish progression, initiating at 34 GPa and completing at 54 GPa. Subsequent electrical resistance measurements unveiled that GeV_4_S_8_ behaves as a semiconductor at room temperature and ambient pressure, transitioning into a nonmetallic conductor at approximately 15 GPa. Remarkably, the transition pressure for the change in the electrical properties is considerably lower than the pressure at which the structural transformation occurs, as indicated by X-ray diffraction data. This implies that the cubic structure undergoes a phase transition from a semiconductor to a nonmetallic conductor under compression at room temperature. Consequently, this alteration in electrical properties cannot be solely ascribed to the structural transformation; instead, it may be associated with the valence electrons in the V_4_ cluster and the compression-induced non-uniform changes among atomic bonds, potentially leading to the Jahn–Teller effect. In conclusion, our comprehensive study, coupled with insights from previous investigations, contributes to a profound and systematic understanding of the behavior of GeV_4_S_8_ under ambient, high-pressure, and low-temperature conditions, as summarized in Figure 9.

As depicted in Figure 9, tentative phase diagrams were formulated by correlating phases with temperature and pressure. Previous investigations primarily delved into cooling conditions at ambient pressure, whereas our present research is centered around compression at room temperature. It is essential to note that our study primarily focuses on the structural and electrical phase transitions without delving into the magnetic properties of GeV_4_S_8_ under compression. Consequently, the determination of phases (structural, electrical, and electromagnetic) remains uncertain under simultaneous alterations in temperature and pressure conditions. Additionally, while we have compared the performance of GeV_4_S_8_ and GaV_4_S_8_ under compression and explored the mechanisms behind the similarities and differences exhibited between these two spinels, there is a need for a more systematic theoretical investigation concentrating on this theme. These observations underscore the trajectory of future research on this material, necessitating a comprehensive approach that encompasses both experimental and theoretical methodologies. Moreover, it emphasizes the significance of the current study, serving as a catalyst and foundational framework for subsequent explorations in this field.

## Supporting Information Available

Crystallographic data of the starting cubic and high-pressure orthorhombic phases of GeV_4_S_8_; Raman band, band frequency under ambient conditions, and Grüneisen parameters of the cubic phase of GeV_4_S_8_; electronic band structure for GeV_4_S_8_; electronic local density of states (LDOS) for GeV_4_S_8_ (PDF)
