# Supplementary material for: Pressure-Induced Changes in the Crystal Structure and Electrical Conductivity of GeV4S8
Source: Chem Mater. 2024 Mar 21;36(7):3128–37. doi: 10.1021/acs.chemmater.3c02488 (PMC11008103; doi:10.1021/acs.chemmater.3c02488)
Supplement: Supplementary file 1 — cm3c02488_si_001.pdf [file cm3c02488_si_001.pdf]

Supporting Information

Pressure-induced Changes in Crystal Structure and Electrical Conductivity of GeV_4S_8

Yuejian Wang*, Zhiwei Shen[#], Dongzhou Zhang[¶], Lin Wang[#], Vladimir Tsurkan[§†], Lilian Prodan[§†], Alois Loidl[§], Bishal B. Dumre[&], Sanjay V. Khare[&]

*Physics Department, Oakland University, Rochester, Michigan 48309, United States
[#]Center for High-Pressure Science (CHiPS), State Key Laboratory of Metastable Materials Science and Technology, Yanshan University, Qinhuangdao, Hebei 066004, China
[¶]Partnership for Extreme Crystallography, University of Hawaii at Manoa, Honolulu, Hawaii 96822, United States
[§]Experimental Physics V, Center for Electronic Correlations and Magnetism, University of Augsburg 86135, Germany
[†]Institute of Applied Physics, Moldova State University, MD-2028 Chisinau, Republic of Moldova
[&]Department of Physics and Astronomy, and Wright Center for Photovoltaics Innovation and Commercialization (PVIC), University of Toledo, Toledo, Ohio 43606, United States

*E-mail: ywang235@oakland.edu

More by Yuejian Wang

http://orcid.org/0000-0001-6663-5912

Table S1: Crystallographic data of the starting cubic and high-pressure orthorhombic phases of GeV_4S_8

Space group		
Pressure	ambient	59.8 GPa
a (Å)<br>b (Å)<br>c (Å)<br>V (Å^3)	9.6576<br><br><br>900.76	6.3577<br>6.7687<br>7.2481<br>311.910
Atomic coordinates	Ga(1): 4a [0, 0, 0]<br>V(1):16e [0.60576, 0.60576, 0.60576]<br>S(1):16e [0.37049, 0.37049, 0.37049]<br>S(2):16e [0.86100, 0.86100, 0.86100]	Ga(1):2a [0, 0, 0]<br>V(1):4d [0, 0.21190, 0.64538]<br>V(2):4c [0.20805, 0, 0.44341]<br>S(1):4d [0, 0.75879, 0.36694]<br>S(11):4c [0.26053, 0, 0.75607]<br>S(2):4d [0, 0.70001, 0.95447]<br>S(21):4c [0.29172, 0, 0.14977]

Table S2: Raman Band, band frequency, $\boldsymbol{\omega_o}$, at ambient condition, and Grneisen parameter, $\boldsymbol{\gamma}$, of the cubic phase of GeV_4S_8. The uncertainties are enclosed in the parenthesis.

Region	Band	$\omega_o\ (cm^{-1})$	$\frac{d\omega}{dP}(\frac{cm^{-1}}{GPa})$	γ
I	A_1	279.1(3.3)	5.4(0.5)	2.05(0.28)
	E	414.2(1.7)	4.4(0.2)	1.12(0.11)
	E	370.7(3.2)	7.1(0.5)	2.01(0.23)
	B_1	346.2(1.5)	4.4(0.2)	1.33(0.12)
II	A_1	318.5(1.7)	2.1(0.1)	0.68(0.05)
	E	440.2(1.6)	2.2(0.1)	0.53(0.03)
	E	418.0(4.1)	3.6(0.1)	0.90(0.08)
	B_1	370.7(1.7)	2.4(0.1)	0.69(0.04)

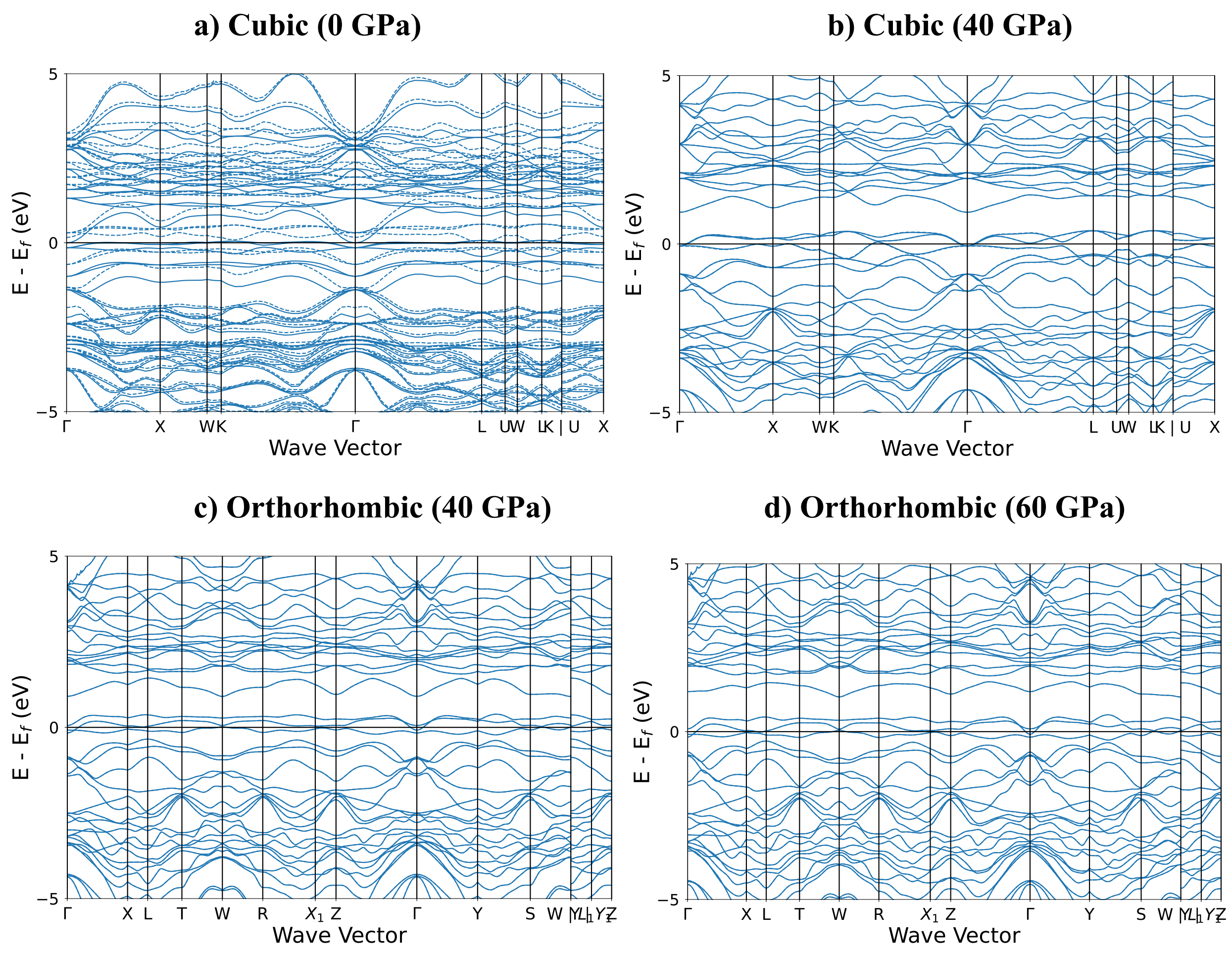

Figure S1: Electronic Band Structure for GeV_4S_8 calculated using GGA in primitive unit cell. The solid line represents spin up whereas dotted lines represent spin down.

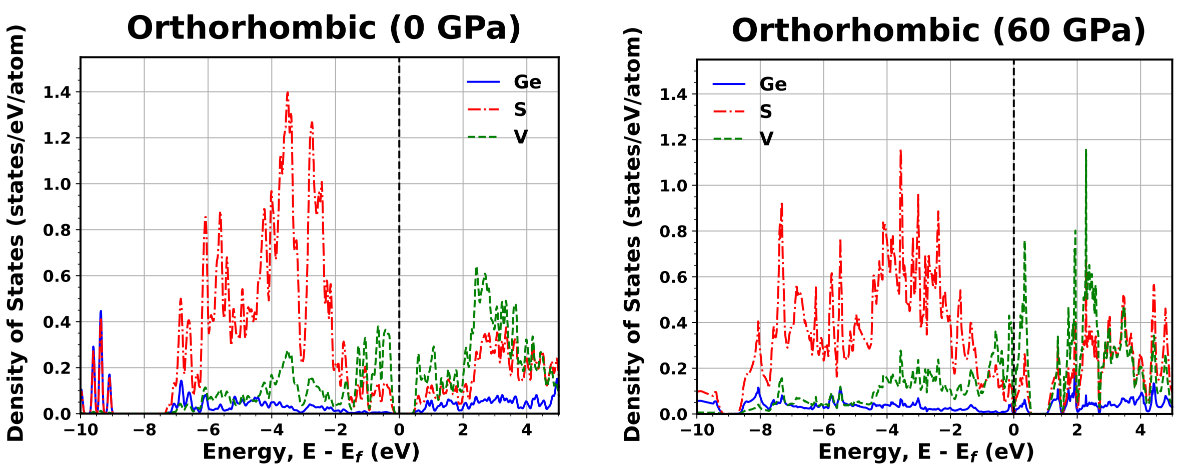

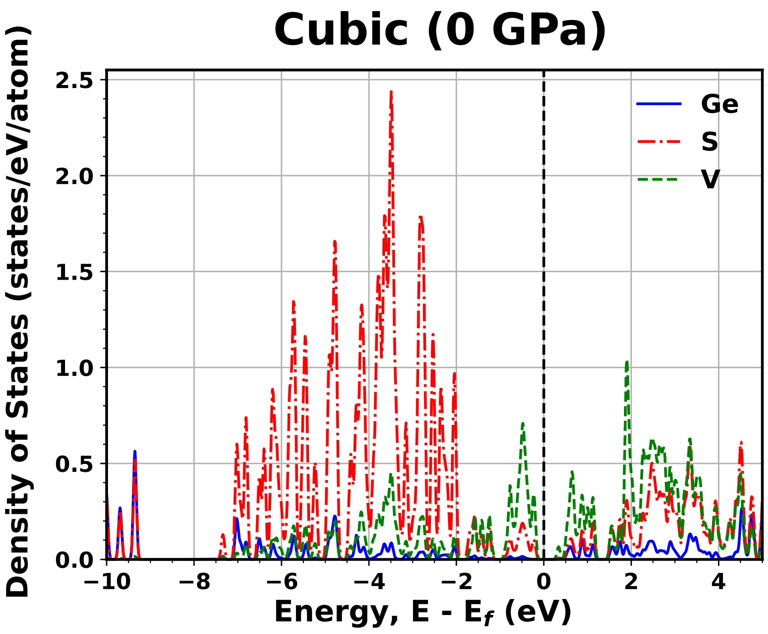

Figure S2. Electronic Local Density of States (LDOS) for GeV_4S_8 calculated using HSE06 in primitive unit cell at different pressures.
